# Bilateral Inflammatory Aural Polyps: A Manifestation of Samter's Triad

**DOI:** 10.1155/2009/464958

**Published:** 2010-02-21

**Authors:** Robert Brobst, Nichole Suss, Stephanie Joe, Saadia Redleaf

**Affiliations:** Department of Otolaryngology-Head and Neck Surgery, University of Illinois at Chicago, Chicago, IL 60612, USA

## Abstract

We report an unusual case of bilateral inflammatory aural polyps in a patient with Samter's triad. This 52-year-old patient had a history of chronic rhinosinusitis with sinonasal polyps, asthma, and aspirin sensitivity, with progressive right-sided hearing loss, otorrhea, and aural fullness. She was found to have bilateral aural polyps, with the larger obstructing lesion on the right. A computed tomography supported these findings and revealed bilateral opacification of the middle ear cleft and mastoid air cells. An initial right tympanomastoidectomy was performed with the specimen histologically resembling a typical sinonasal polyp. We speculate that this patient's middle ear polyposis is secondary to the inflammatory changes of Samter's triad. This has not been described previously in the literature.

## 1. Introduction

Samter's triad clinically presents as chronic rhinosinusitis (CRS) with nasal polyps, aspirin sensitivity, and bronchial asthma in an adult nonatopic patient [1]. Investigations into the pathophysiology of Samter's triad have found the etiology to be unclear, but arachidonic acid metabolites and a heightened host tissue responsiveness are common to these patients. A relatively decreased protective prostaglandin E2 effect and increased inflammatory cysteinyl leukotriene levels have been found in previous studies [2, 3]. Histopathological evaluation of sinonasal polyps and bronchial tissue reveal abundant eosinophil infiltration and degranulated mast cells within the upper and lower airways [4]. In these patients manifesting both CRS and asthma, optimal treatment outcomes for either site are dependent on adequate treatment of the other. Medical management with topical nasal, inhaled, and/or oral steroids with avoidance of acute aspirin and NSAID ingestion is the mainstay of treatment. However, multiple surgical interventions for treatment of the sinuses and removal of obstructing polyps are usually required. In severe cases, aspirin desensitization has been used with symptomatic improvement [5].

Aural polyps usually present as chronic otorrhea causing lesser complaints of hearing loss, aural bleeding, otalgia, and vertigo. Typically, polyps are unilateral, protruding through the tympanic membrane from the middle ear cleft and completely occluding the external auditory canal [6, 7]. Chronic otitis media and cholesteatoma are the most frequent underlying etiology of polyps, with rare systemic inflammatory infections and lymphoproliferative processes as alternative causes. Histopathologically, polyps demonstrate chronic inflammatory cells including eosinophils, neutrophils, multinucleated giant cells, and plasma cells in a variable stroma. Up to 35% are associated with underlying cholesteatoma, often without reliable prognosticators. Therefore treatment recommendations differ between simple aural polypectomy and mastoidectomy, with proponents of the latter considering aural polyps as unsafe disease [6]. We present a case of aural polyps secondary to Samter's triad. Surgical intervention was unsuccessful and hearing loss improved with steroid administration.

## 2. Case Report

**Figure 1:**
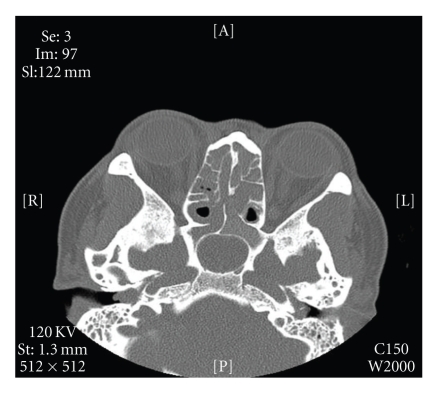
Axial computed tomographic scan demonstrates bilateral aural polyps with thickened sinonasal mucosa.

A 52-year-old female, with history of Samter's triad, treated twice previously with functional endoscopic sinus surgery for recurrent nasal polyposis, returned for evaluation of ongoing right otalgia, otorrhea, and hearing loss. On exam, purulent drainage and a large right aural polyp were found completely obstructing tympanic membrane visualization. The left showed an asymptomatic posterior superior polyp. Medical management was continued with amoxicillin and ciprodex otic drops, and a right biopsy was taken. Results were consistent with pyogenic granuloma. Audiogram identified a symmetric 20 to 30 decibel conductive hearing loss with a flat tympanogram bilaterally. Computed tomography showed well-developed mastoid air cells bilaterally with evidence of bilateral soft tissue disease and polyps greater on the right than on the left. No evidence of bony erosion concerning cholesteatoma was seen (Figure 1).

**Figure 2:**
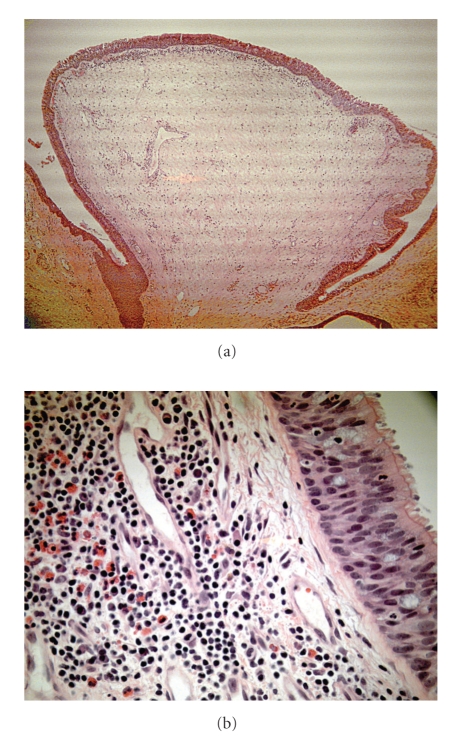
Low-power histopathology of the inflammatory otic polyp (a) with high-power (b) demonstration of eosinophils underlying surface pseudostratified columnar epithelium with cilia.

**Figure 3:**
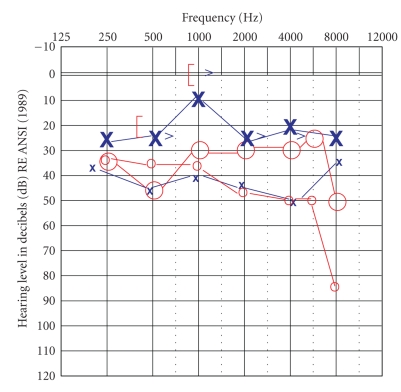
Bilateral pure tone audiogram post steroid infusion. Small X's and O's indicate pretreatment thresholds.

The patient then underwent a right tympanomastoidetcomy with numerous polyps identified during dissection within the sinodural angle, epitympanum, and the remaining middle ear space. No evidence of keratin debris was found during the dissection. The histopathology was reported as mucosa containing inflammatory otic polyps with eosinophils and Charcot-Lyden crystals. Findings consistent with cholesteatoma were not observed (Figure 2). Postoperatively, the patient's middle ear mucosal hypertrophy recurred. Bilateral pressure-equalization tubes were placed and 0.5 ml of triamcinolone (40 mg/cc) was instilled every three to four weeks. The patient used steroid and antibiotic otic drops and had one course of prednisone (60 mg) taken by mouth for seven days. After six infusions and one prednisone course by mouth, pure tone thresholds improved (Figure 3).

Several months after the cessation of topical and oral steroid treatment, the patient's pressure equalization tubes both extruded and the middle ear obstructive pathology returned, with concomitant conductive hearing losses. She currently experiences cycles of replacement of her extruded tympanostomy tubes, topical steroid treatments, and spontaneous extrusion of her tubes.

## 3. Discussion

Evidence of bilateral chronic otitis media is present in 50% of patients with aural polyps, but bilateral polyps are rarely described in the literature [6, 8]. In this patient's left ear, no history of chronic disease was present in association with the posterior superior polyp. Additionally, surgical pathology evaluation of the right polyps appeared consistent with those typically removed from the nasal cavity in chronic rhinosinusitis. 

 The pseudostratified columnar ciliated mucosal lining of the nasal cavities and anterior inferior middle ear cleft is similar [9]. Despite common histology and close anatomic relationship, polypoid changes are not described routinely in the Eustachian tubes or middle ear cleft in patients with advanced CRS with nasal polyp disease. With the systemic inflammatory changes associated with Samter's triad, bilateral presentation, and consistent histology, it is plausible that these findings are secondary to an otic manifestation of this disease. Surgical management proved unsuccessful in this patient. Instead, intratympanic and oral steroid administration yielded improvement in auditory thresholds.
